# Temporal evidence fusion evaluation method considering time sequence variation trend

**DOI:** 10.1038/s41598-025-10687-7

**Published:** 2025-07-11

**Authors:** Sunan Zhang, Yanxin Gao, Yuanchao Kou, Qichao Guo

**Affiliations:** 1 Engineering Training Center, Taiyuan Institute of Technology, Taiyuan, 030008 China; 2 Strengthening Foundation Institute, Shanxi Institute of Energy, Taiyuan, 030604 China

**Keywords:** Evidence theory, Time sequence trend, Temporal variation factor, Evidence combination rule, Fusion evaluation, Information technology, Computer science

## Abstract

Aiming at the temporal information fusion evaluation problem, a temporal evidence fusion evaluation method was proposed by considering the time sequence trend based on evidence theory to fully reflect the dynamic trend of temporal information and the influence of the time factor on fusion evaluation. Firstly, the trend of temporal evidence sequence was integrated into fusion evaluation. The temporal variation factor of the proposition was defined by analyzing the sequence of changes in evidence to measure the dynamic of the proposition. Then, conflict information between evidence was interpreted as the temporal variation information of propositions. An evidence combination rule integrating temporal variation factors of propositions was proposed in this paper. Finally, the proposed method was used to fuse and evaluate the temporal evidence. The consecutive periodic health assessment data were utilized to validate the fusion results. Numerical examples show that the discrepancy between the combined BPAs obtained using the proposed method is significantly larger, while the associated uncertainty is notably reduced. The proposed method is capable of handling conflicting information within a temporal information sequence and obtaining a reasonable fusion evaluation result by combining the temporal variation trend of propositions. It provides an idea for information fusion evaluation considering the time sequence trend.

## Introduction

Multi-source information fusion is one of the important research topics in fault diagnosis^1,2^, navigation^3^, and fusion evaluation^4,5^. Its essence is to combine the data and knowledge of various information sources according to the redundant or complementary information in space or time according to specific standards, to obtain a consistent interpretation or description of the object under test, so that the information system has better performance compared with the system composed of each subset^6–8^. Especially in the multi-index evaluation, the evaluation results can be obtained much more accurately and comprehensively through multi-source information fusion. In practical application, Rough set theory^4^ and DS evidence theory^9^ are standard and effective methods for multi-source information fusion.

The rough set theory is a mathematical tool extensively utilized in uncertainty data analysis. The theory has been successfully applied to the fields of attribute selection^10,11^ and attribute reduction^12,13^, thereby providing robust support for the efficient fusion of multi-source data. However, the classical rough set model relies on equivalence relations, which are overly restrictive and significantly limit the applicability of rough sets. As an innovative approach for fusing multi-source information, the multi-granularity rough set model enhances the capacity of multi-source information systems to address uncertainty-related issues.

DS evidence theory has been widely used can effectively solve some uncertain information in the location environment without prior information. At present, most evidence-theory-based uncertain information fusion focuses on integrating information from multiple uncertain sources. This process involves fusing uncertain information provided by various information sources at a specific moment using evidence theory, which can be termed spatial evidence fusion. In the process of spatial evidence fusion using DS evidence theory, various group decision-making rules are employed to enhance the accuracy and reliability of the fusion results^14^. Song et al.^15^ used evidence theory and intuitionistic fuzzy sets to evaluate sensor dynamic reliability. The evidence discounting was applied to improve Dempster’s combination rule. Zhao et al.^16^ proposed a two-perspective approach based on evidence theory to evaluate the reliability of sources of evidence. The proposed two-perspectives approach can relieve high conflict and improve the accuracy of evidence theory-based classification problems by generating discounting rules. Li et al.^17^ proposed an improved DS evidence theory combined rough set theory to identify various operation states of the fire control system. An improved conflict evidence synthesis method is proposed to solve the impact of conflicts in the existing DS evidence theory. Liu^18^ improved the representation of the voting unit’s decision and the fusion rule by DS evidence theory. The measurement error and decision preference were considered in the voting unit. Sun^19^ used DS evidence theory to evaluate the safety status in the process of coal mine gas safety evaluation. The idea of assigning weight and conflict allocation coefficient was presented to fuse high-conflict evidence and prevent the loss of the effective information of the original evidence. Driss et al.^20^ applied Dempster-Shafer theory to fuse the results of five per-trained convolutional neural networks for the diagnosis of pneumonia from chest X-ray images. There is no problem in considering the multi-time information evaluation. But for the complex multi-index evaluation system, the time factor has a great influence on the fusion evaluation results. It is difficult to complete the fusion evaluation comprehensively at a specific moment, and it is necessary to fuse evidence in a time sequence, namely, temporal fusion evaluation. Based on the research on the spatial evidence fusion method, temporal evidence fusion is gradually attracting researcher’s attention. Kushwah et al.^21^ introduced temporal information into evidence theory to recognize indoor activity by a series of passive sensors. Fan et al.^22^ evaluated the reliability of temporal evidence based on the correlation between temporal evidence variables. Zhang et al.^23^ proposed a time-domain evidence fusion method involving the time sequence of the decision-maker. This approach fully considers the influence of dynamic features and temporal factors in time-domain information fusion on the fusion process. Wang et al.^24^ proposed an improved method of evidence theory to monitor the state of a gas regulator station. In order to fuse the time-domain information dynamically, the time attenuation factor and the relative conflict factor were combined to modify the evidence and reduce the accumulated error.

Through the above analysis, it can be seen that most of the studies are carried out on the basis of spatial evidence fusion, without sufficient consideration of the temporal variation between evidence, and the dynamic variation trend of propositions is rarely involved. In the temporal evidence fusion evaluation, the temporal variation trend of each proposition has an important influence on the final fusion evaluation result. To solve the above problems, a temporal evidence fusion evaluation method with consideration of the time sequence trend of the proposition was proposed in this paper. Temporal variation factors of propositions were defined to measure the dynamic trend of propositions. In the process of fusion, the conflict information between evidence was interpreted as the temporal variation information of propositions, and this information was allocated and processed according to the temporal variation factors of propositions, to realize the fusion evaluation of temporal evidence considering the temporal variation trend. Generally, the major contributions of our paper can be summarized as follows:

1. The trend of the temporal evidence sequence was incorporated into the fusion evaluation process.
2. Conflict information between evidence sources was reinterpreted as temporal variation information of propositions.
3. A novel evidence combination rule that integrates temporal variation factors of propositions was proposed.

The rest paper is organized as follows: A brief introduction of evidence theory is given in Sect. 2. The temporal evidence fusion evaluation method is proposed in Sect. 3. The experimental results and discussion are shown in Sect. 4 and the conclusion is drawn in Sect. 5.

## DS evidence theory

Evidence theory is a complete theory proposed by Dempster and Shafer to efficiently solve uncertain problems^25,26^. The uncertainty is studied based on a set of mutually exclusive and exhaustive events or elements about a problem domain. The perfect set is called the frame of discernment *Θ*, which can be expressed as *Θ*={*θ*_1_, *θ*_2_, ···*θ*_*j*_ ···, *θ*_*N*_}. Where, *θ*_*j*_ is an event or element in *Θ*, and *N* is the number of events or elements. The set 2^*Θ*^ composed of all the possible subsets of *Θ* is called the power set of *Θ*. It can be expressed as 2^*Θ*^={*Φ*,{*θ*_1_}, {*θ*_2_}, ···, {*θ*_*N*_}, {*θ*_1_, *θ*_2_}, {*θ*_1_, *θ*_3_}, ···, *Θ*}. Suppose A is the arbitrary nonempty subset of *Θ*, and *Φ* is the empty set. If the mapping function $m:{2^\Theta } \to [0,1]$ could satisfy:

1. *m*(*Φ*) = 0;
2. $$\sum\nolimits_{A \subseteq \Theta } {m(A) = 1};$$
3. *m*(*A*) ≥ 0

Then *m* is called a basic probability assignment (BPA) of *Θ*^27^. m(A) refers to the degree of belief that is assigned to the subset *A*. If *m*(*A*) > 0, then *A* is called a focal element.

The combination rule of DS evidence theory is to combine different evidence in a certain way to obtain a more comprehensive joint BPA.

Suppose *m*_1_ and *m*_2_ are two BPAs of *Θ*. The combination rule of DS evidence theory can be described as follows:

1 $$\left\{ {\begin{array}{*{20}{c}} {m(A)={m_1} \oplus {m_2}(A)=\frac{{\sum\limits_{{{A_i} \cap {B_j}=A}} {{m_1}({A_i}){m_2}({B_j})} }}{{1 - K}}} \\ {K=\sum\limits_{{{A_i} \cap {B_j}=\phi }} {{m_1}({A_i}){m_2}({B_j})} } \end{array}} \right.$$

where *K* is the conflict coefficient, which represents the total conflict between the two independent evidence; *m*_1_(·) and *m*_2_(·) are the evidence from two different sources; *A*_*i*_ and *B*_*j*_ are the two different propositions.

To generalize to multiple evidence fusion, let *m*_1_, *m*_2_,…, *m*_*n*_ are *n* mutually independent BPAs. The joint BPA^28^ can be expressed as follows:

2 $$\left\{ {\begin{array}{*{20}{c}} {m(A)=\frac{{\sum\limits_{{ \cap {A_i}=A}} {\prod\limits_{{j=1}}^{n} {{m_j}({A_i})} } }}{{1 - K}}} \\ {K=\sum\limits_{{ \cap {A_i}=\phi }} {\prod\limits_{{j=1}}^{n} {{m_j}({A_i})} } } \end{array}} \right.$$.

## Temporal evidence fusion evaluation method

### The temporal variation factor of proposition

In the process of temporal evidence fusion, the influence of evidence sequence on the fusion result is considered. In essence, the methods to assign different weights of evidence through correlation, distance, and information entropy among temporal evidence belong to the spatial evidence analysis method. These methods do not pay attention to the dynamic trend of temporal evidence. However, it is not only necessary to consider the relationship between evidence at different moments, but also the dynamic variation trend of propositions at different moments in the temporal evidence fusion evaluation. The variation trend of propositions can reflect the development bias of evaluation objects to a great extent. For example, in the patient health integration evaluation, the change of evaluation indicators over time has an important influence on the final evaluation result. If the patient’s health status deteriorates gradually over time, the severity of the disease should be reflected in the final fusion evaluation results. On the contrary, the results of the fusion evaluation should reflect the improvement of the disease. The temporal variation factor of proposition is defined to describe the change of proposition at different times.

Suppose *m*_1_, *m*_2_, ···, *m*_*n*_ are *n* BPAs in the frame of discernment *Θ*={*θ*_1_, *θ*_2_, ···, *θ*_*N*_}. $Dif_{{ij}}^{k}$ is the difference between *m*_*j*_(*A*_*k*_) and *m*_*i*_(*A*_*k*_), which can be defined as follows:

3 $$Dif_{{ij}}^{k}={m_j}({A_k}) - {m_i}({A_k})$$.

where *i*, *j* are the serial numbers of propositions, $1 \leqslant i,j \leqslant n$; *A*_*k*_ is the *k*th proposition, $1 \leqslant k \leqslant p$; and *p* is the number of propositions.

Construct the difference matrix ***Dif***_*k*_ of the propositions as below:

4 $${\mathbf{Di}}{{\mathbf{f}}_k}=\left[ {\begin{array}{*{20}{c}} {Dif_{{11}}^{k}}&{Dif_{{12}}^{k}}& \cdots &{Dif_{{1i}}^{k}}& \cdots &{Dif_{{1n}}^{k}} \\ {Dif_{{21}}^{k}}&{Dif_{{22}}^{k}}& \cdots &{Dif_{{2i}}^{k}}& \cdots &{Dif_{{2n}}^{k}} \\ \vdots & \vdots &{}& \vdots &{}& \vdots \\ {Dif_{{j1}}^{k}}&{Dif_{{j2}}^{k}}& \cdots &{Dif_{{ji}}^{k}}& \cdots &{Dif_{{jn}}^{k}} \\ \vdots & \vdots &{}& \vdots &{}& \vdots \\ {Dif_{{n1}}^{k}}&{Dif_{{n2}}^{k}}& \cdots &{Dif_{{ni}}^{k}}& \cdots &{Dif_{{nn}}^{k}} \end{array}} \right]$$.

If the temporal growth of proposition BPA is concerned, the total variation of proposition *A*_*k*_ is:

5 $$Sup({A_k})=\sum\limits_{\begin{subarray}{l} j>i \\ i=1,2, \cdots ,n - 1 \\ j=2,3, \cdots ,n \end{subarray} } {Dif_{{ij}}^{k}}$$.

Then the total variation vector of the proposition can be expressed as ***Sup***=[*Sup*(*A*_1_),*Sup*(*A*_2_),···,*Sup*(*A*_*p*_)]. Normalize the total variation of proposition *A*_*k*_ as follows:

6 $$\tilde {S}up({A_k})=\frac{1}{{1+{e^{ - (\alpha (Sup({A_k})+\hbox{min} ({\mathbf{Sup}})))}}}}$$

where $\tilde {S}up({A_k})$ is the normalized variable of *Sup*(*A*_*k*_); *α* is the normalized linear coefficient of the total variation of propositions; min(***Sup***) is the minimum value of ***Sup***.

The temporal variation factor *σ*_*k*_ of the proposition *A*_*k*_ can be expressed as:

7 $${\sigma _k}={{\tilde {S}up({A_k})} \mathord{\left/ {\vphantom {{\tilde {S}up({A_k})} {\sum\limits_{{r=1}}^{p} {\tilde {S}up({A_r})} }}} \right. \kern-0pt} {\sum\limits_{{r=1}}^{p} {\tilde {S}up({A_r})} }}$$.

Obviously, 0 < *σ*_*k*_ < 1, the greater *σ*_*k*_, indicating that the BPA of proposition *A*_*k*_ increases with the increase of time series. The closer *σ*_*k*_ is to 0, it indicates that the BPA of proposition *A*_*k*_ does not change with time series. The closer *σ*_*k*_ is to 1, the BPA of almost only *A*_*k*_ increases with time series for all propositions.

Similarly, if we pay more attention to the temporal reduction of proposition BPA, formula (5) can be adjusted as:

8 $$Sup({A_k})=\sum\limits_{\begin{subarray}{l} j<i \\ i=2,3, \cdots ,n \\ j=1,2, \cdots ,n - 1 \end{subarray} } {Dif_{{ij}}^{k}}$$.

### Temporal evidence combination rule

In evidence theory, there are the following problems in the practical application of evidence combination rules:

① According to Formula (2), when the BPA of a proposition in evidence is 0, regardless of the BPA of the proposition in other evidence, the combined BPA is 0;
② In the case of highly conflicting evidence, evidence invalidation will occur in the rule of evidence synthesis, that is, the fusion results are inconsistent with the facts. If *K* = 1, it indicates that the two evidence completely conflict. According to Formula (2), the synthesis formula is invalid at this time.

In view of the above problems, the applicability of combination rules of evidence theory is analyzed. Some studies believe that the main reason for the above problems is the problems in the data obtained from evidence, especially the conflicts between evidence, mainly caused by unreliable sources of evidence. Therefore, evidence theory improves the weight of evidence according to the importance of evidence to deal with conflict. In such cases, most assessments of the weight of evidence are based on “majority rule”. If any evidence is supported by most of the other evidence, a higher weight can be given to this evidence. In the case of only two pieces of evidence, if there is a great conflict between them, then at least one of the two pieces of evidence is considered to be unreliable. Other studies believe that the above problems are caused by the rule of evidence combination. When conflict evidence is obtained, it is necessary to analyze the conflict. The conflict evidence still contains some useful information, which needs to be rationally distributed according to the rules. By summarizing and analyzing the conflict coefficient allocation methods, a universal fusion formula can be obtained:

9 $$m(A)=\sum\limits_{{B \cap C=A}} {{m_1}(B){m_2}(C)+K \cdot \delta (A,m)}.$$

where *A*,*B*,*C*∈2^*Θ*^; *δ*(*A*,*m*) is the propositional weight, $\sum\limits_{{A \subseteq \Theta }} {\delta (A,m)=1}$.

In time domain evidence fusion evaluation, evidence comes from different moments and has time-series variation. To some extent, the conflicts between evidence represent the changes of evidence to time series. The greater the conflict between evidence, the greater the variation of evidence over time series. The less the conflict between evidence, the less the variation of evidence over time series. The conflicting information of evidence is the time series variation information. Therefore, conflict evidence is not necessarily unreliable, and its uncertainty may be smaller and contain more information about temporal variation than evidence with less conflict. If the fusion is carried out according to the principle of “majority rule”, the evidence that changes greatly may be ignored and the information may be lost. Therefore, in time domain evidence fusion evaluation, the variation of propositions in temporal evidence should be fully considered. In the process of fusion, the change information is allocated reasonably according to the temporal variation factor of the proposition.

Referring to Formula (9), the temporal variation information is allocated in this paper. In the temporal evidence fusion evaluation, the temporal variations of propositions are different. Because of the temporal variation information between evidence, the temporal variation information is allocated according to the variation trend of different propositions. The fusion formulas are shown in Formula (10) and Formula (11).

10 $$m(\Phi )=0$$

11 $$m(A)=\sum\limits_{{ \cap {A_i}=A}} {\prod\limits_{{j=1}}^{n} {{m_j}({A_i})+{\sigma _A}\sum\limits_{{ \cap {A_s}=\phi }} {\prod\limits_{{l=1}}^{n} {{m_l}({A_s})} } } }$$

where *σ*_*A*_ is the temporal variation factor of proposition *A*.

The first part of Formula (11) is the consistency information of temporal evidence, and the second part is the allocation of conflict information within the temporal evidence. In the newly proposed fusion formula, the conflicts between evidence are treated as indicators of temporal change information. The existing conflicts between evidence are redistributed by the temporal variation factors associated with propositions, thereby leveraging the conflict information more effectively. This novel fusion rule not only considers the consistency of information but also fully exploits the temporal variations in evidence, thus improving the reliability of temporal evidence fusion.

### Evidence fusion evaluation considering Temporal variation trend

Suppose *m*_1_, *m*_2_, ···, *m*_*n*_ are *n* BPAs in the frame of discernment *Θ*={*θ*_1_, *θ*_2_, ···, *θ*_*N*_}. The steps of the temporal evidence fusion evaluation method with consideration of the time sequence trend of the proposition proposed in this paper are as follows:

Step 1: Calculate the difference matrix of each proposition between evidence according to formula (3) and formula (4).

Step 2: Determine the temporal variation tendency of a proposition according to the temporal evidence fusion evaluation object. If the temporal growth of proposition BPA is concerned, the total variation of the proposition is calculated according to Formula (5). If the temporal decrease of proposition BPA is concerned, the total variation of a proposition is calculated according to Formula (8).

Step 3: Calculate the temporal variation factor of each proposition according to formula (6) and formula (7).

Step 4: According to the temporal variation factor of each proposition, fuse the time-domain evidence as Formula (10) and Formula (11), and obtain the final fusion evaluation result.

In the process of fusion evaluation, the temporal variation information between evidence is considered, and the fusion evaluation result is related to the temporal variation between evidence. Therefore, the method proposed in this paper does not satisfy the commutative law and the associative law.

## Results and discussion

**Fig. 1:**
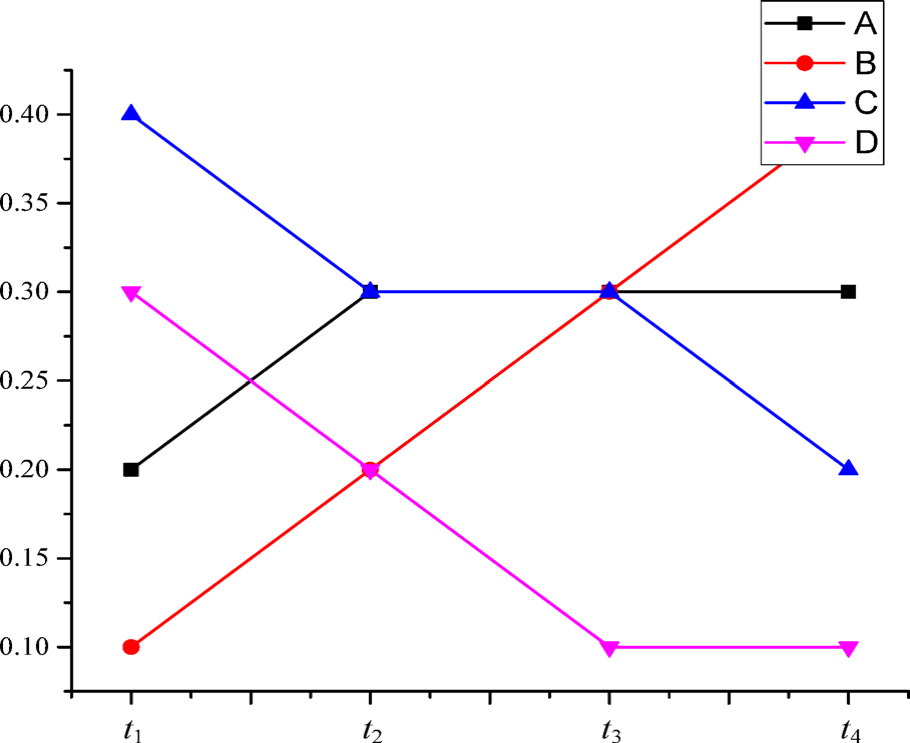
The time sequence trends of {*A*}, {*B*}, {*C*}, and {*D*}.

A health evaluation system evaluates the health of the same object in four consecutive sequential evaluation periods. Categories of evaluation objects are *A*, *B*, *C*, and *D*. Therefore, the evaluation results in four sequential evaluation periods can be converted into BPAs at *Θ* ={*A*, *B*, *C*, *D*}, as shown in Table 1. Time sequence trends of {*A*}, {*B*}, {*C*}, and {*D*} are shown in Fig. 1.

**Table 1:** BPAs obtained in different evaluation periods.

BPA	A	B	C	D
*t*_1_:*m*_1_(·)	0.2	0.1	0.4	0.3
*t*_2_:*m*_2_(·)	0.3	0.2	0.3	0.2
*t*_3_:*m*_3_(·)	0.3	0.3	0.3	0.1
*t*_4_:*m*_4_(·)	0.3	0.4	0.2	0.1

Fuse the evaluation information obtained in different evaluation periods by the temporal evidence fusion evaluation method proposed in this paper. The fusion process is as follows:

1. Calculate the difference matrices ***Dif***_*A*_, ***Dif***_*B*_, ***Dif***_*C*_, ***Dif***_*D*_ of propositions {*A*}, {*B*}, {*C*}, {*D*}:
  $${\mathbf{Di}}{{\mathbf{f}}_A}=\left[ {\begin{array}{*{20}{c}} {0.0}&{0.1}&{0.1}&{0.1} \\ { - 0.1}&{0.0}&{0.0}&{0.0} \\ { - 0.1}&{0.0}&{0.0}&{0.0} \\ { - 0.1}&{0.0}&{0.0}&{0.0} \end{array}} \right]$$
  $${\mathbf{Di}}{{\mathbf{f}}_B}=\left[ {\begin{array}{*{20}{c}} {0.0}&{0.1}&{0.2}&{0.3} \\ { - 0.1}&{0.0}&{0.1}&{0.2} \\ { - 0.2}&{ - 0.1}&{0.0}&{0.1} \\ { - 0.3}&{ - 0.2}&{ - 0.1}&{0.0} \end{array}} \right]$$
  $${\mathbf{Di}}{{\mathbf{f}}_C}=\left[ {\begin{array}{*{20}{c}} {0.0}&{ - 0.1}&{ - 0.1}&{ - 0.2} \\ {0.1}&{0.0}&{0.0}&{ - 0.1} \\ {0.1}&{0.0}&{0.0}&{ - 0.1} \\ {0.2}&{0.1}&{0.1}&{0.0} \end{array}} \right]$$
  $${\mathbf{Di}}{{\mathbf{f}}_D}=\left[ {\begin{array}{*{20}{c}} {0.0}&{ - 0.1}&{ - 0.2}&{ - 0.2} \\ {0.1}&{0.0}&{ - 0.1}&{ - 0.1} \\ {0.2}&{0.1}&{0.0}&{0.0} \\ {0.2}&{0.1}&{0.0}&{0.0} \end{array}} \right]$$
2. Assuming that the health assessment system pays more attention to the temporal growth of proposition BPA, then the total variations *Sup*(*A*), *Sup*(*B*), *Sup*(*C*), *Sup*(*D*) of proposition {*A*}, {*B*}, {*C*}, {*D*} are: *Sup*(*A*) = 0.3, *Sup*(*B*) = 1.0, *Sup*(*C*)=-0.6, *Sup*(*D*)=-0.7.
3. Let the normalized linear coefficient *α* = 3 of the total variations of the propositions. Then, total variations after normalization which retain two decimal places are: $\tilde {S}up(A) \approx 0.23$, $\tilde {S}up(B) \approx 0.71$, $\tilde {S}up(C) \approx 0.02$, $\tilde {S}up(D) \approx 0.01$.
4. Calculate the temporal variation factors of propositions {*A*}, {*B*}, {*C*}, and {*D*} as formula (7), and two digits after the decimal point are reserved respectively: *σ*_*A*_ = 0.24, *σ*_*B*_ = 0.73, *σ*_*C*_ = 0.02, *σ*_*D*_ = 0.01.
5. Fuse the evaluation information obtained in different evaluation periods by the temporal evidence combination rule defined in formula (10) and formula (11). The results are as follows: *m*({*A*}) = 0.24, *m*({*B*}) = 0.72, *m*({*C*}) = 0.03, *m*({*D*}) = 0.01.

As shown in Fig. 1, the BPA of proposition *B* increases gradually with the increase of the evaluation period in this example. Compared with other propositions, the temporal variation factor of proposition *B* should be the largest. The temporal variation factor of proposition *B* is calculated as *σ*_*B*_ = 0.73, which is larger than that of other propositions. It is consistent with the analysis results. In the fusion process, although the BPAs of Proposition *A*, Proposition *C*, and Proposition *D* in the first three evaluation periods are large, the temporal variation of Proposition *A* is small and the temporal variations of Proposition *C* and Proposition *D* are gradually reduced. Therefore, the BPAs of Proposition *A*, Proposition *C*, and Proposition *D* after fusion should be smaller than that of Proposition *B*. The calculated fusion evaluation result is *m*({*B*}) = 0.72, which is the highest among all the fusion evaluation results. It is consistent with the analysis results.

In order to facilitate comparative analysis, the fusion results obtained by other methods and the fusion evaluation results obtained by this method are presented in Table 2.

**Table 2:** Fusion evaluation results obtained by different methods.

Method	{A}	{B}	{C}	{D}	Θ
Dempste’s rule	0.3500	0.1500	0.4600	0.0400	0.0000
Yager’s rule^29^	0.0054	0.0024	0.0072	0.0006	0.0000
Murphy’s method^30^	0.3100	0.2100	0.4300	0.0500	0.0000
Deng’s method^31^	0.3100	0.2100	0.4300	0.0500	0.0000
DCre-Weight^32^	0.2400	0.2300	0.2700	0.1600	0.1000
Method proposed in this paper(*α* = 3)	0.2400	0.7200	0.0300	0.0100	0.0000

Table 2 shows that the combination rules of Dempster, Yager, Murphy, and Deng all have the highest BPA in Proposition *C*. DCre-weight method has the highest BPA in Proposition *A*. These methods only consider the spatial information of evidence. As Proposition *A* and Proposition *C* in different evidence have higher BPA, the fusion evaluation result is *A* or *C*. Moreover, there is little difference in the combined BPA of each proposition, and the uncertainty of the propositions is strong after the fusion evaluation. The method proposed in this paper takes temporal and spatial information into account in the process of fusion evaluation. As the BPA of proposition *B* gradually increases with the time series of the evaluation period, its temporal variation factor is the largest. The temporal variation information assigned to proposition *B* is also the largest. Therefore, its combined BPA is the largest. Compared with the combined BPA with other propositions, the difference is large, and the uncertainty is smaller than other combination rules. Therefore, the proposed method can better reflect the characteristics of temporal fusion and the influence of the temporal variation trend of the proposition on the fusion evaluation results.

**Fig. 2:**
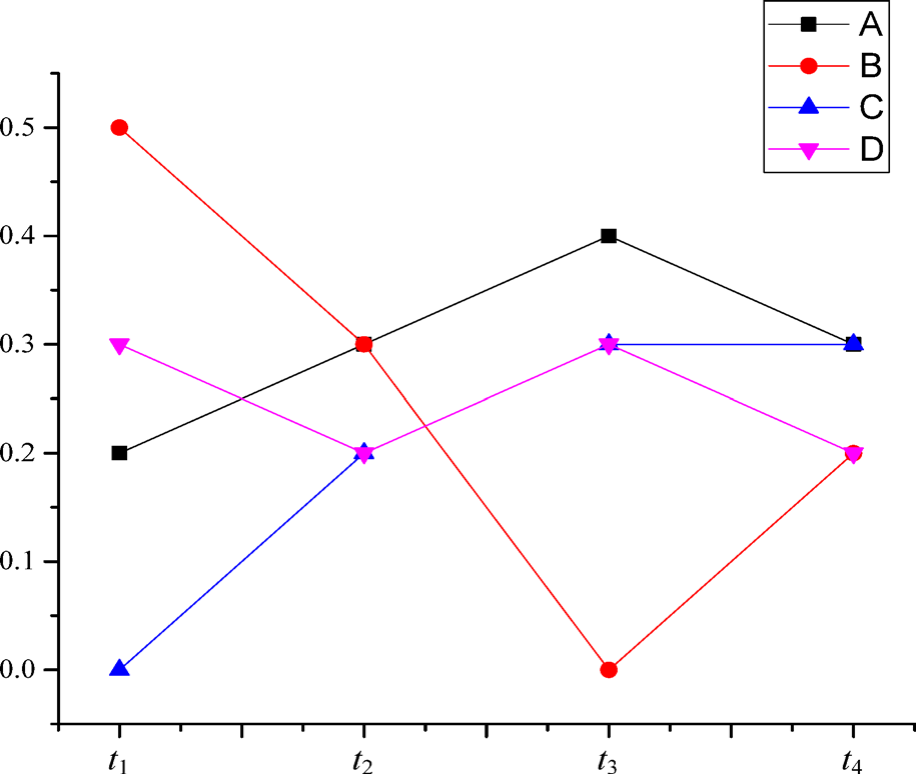
The time sequence trends of {*A*}, {*B*}, {*C*}, and {*D*}.

In order to analyze the effectiveness of the method proposed in this paper in high-conflict evidence fusion evaluation, the BPAs obtained in four sequential evaluation periods as shown in Table 3 are selected for fusion evaluation. The time sequence trends of {*A*}, {*B*}, {*C*}, and {*D*} are shown in Fig. 2.

**Table 3:** BPAs obtained in different evaluation periods.

BPA	{A}	{B}	{C}	{D}
*t*_1_:*m*_1_(·)	0.2	0.5	0.0	0.3
*t*_2_:*m*_2_(·)	0.3	0.3	0.2	0.2
*t*_3_:*m*_3_(·)	0.4	0.0	0.3	0.3
*t*_4_:*m*_4_(·)	0.3	0.2	0.3	0.2

The combination rules of Dempster, Yager, Murphy, Deng, DCre-weight, and the method proposed in this paper are used for fusion evaluation, and the results are shown in Table 4.

**Table 4:** Fusion evaluation results obtained by different methods.

Method	{A}	{B}	{C}	{D}	Θ
Dempste’s rule	0.6700	0.0000	0.0000	0.3300	0.0000
Yager’s rule	0.0072	0.0000	0.0000	0.0036	0.0000
Murphy’s method	0.4600	0.2200	0.1000	0.2200	0.0000
Deng’s method	0.4800	0.2100	0.1000	0.2100	0.0000
DCre-Weight	0.2500	0.2300	0.1200	0.2500	0.1500
Method proposed in this paper(*α* = 3)	0.1890	0.0020	0.7730	0.0360	0.0000

Table 4 shows that the BPA of Proposition *C* is 0 in the first evaluation period, and that of Proposition *B* is 0 in the third evaluation period. The Proposition *C* is completely negated in the first evaluation period and the Proposition *B* is completely negated in the third evaluation period. The combined BPAs of Dempster’s rule and Yager’s method are 0 regardless of the BPAs of Proposition *B* and Proposition *C* in other evaluation periods. The combination rules of Dempster, Yager, Murphy, Deng, and DCre-weight all have the highest BPA in Proposition *A*. The uncertainty of the propositions is strong after fusion evaluation by the methods of Murphy, Deng, and DCre-weight. After the method proposed in this paper considers the time series variation trend, proposition *C* gradually increases with the time series growth of the evaluation periods. As shown in Fig. 2, although the BPA of proposition *C* remains constant from the third assessment period to the fourth assessment period, its temporal variation tends to increase. As the other propositions have no continuous trend of growth, the final fusion evaluation result is *C*. It shows that the proposed method can reflect the influence of time factor and the dynamic trend of temporal information on the fusion evaluation, and realizes the temporal evidence fusion evaluation considering the temporal trend.

## Conclusions

The temporal evidence fusion method considering the time sequence trend of propositions based on evidence theory is studied in this paper. The temporal variation trend of propositions is described by defining temporal variation factors. In the process of evidence fusion, the conflict between evidence is analyzed. In temporal evidence fusion considering time sequence trend, the conflict information between evidence is interpreted as temporal variation information of propositions, and the temporal variation information is allocated by temporal variation factors. The experimental results show that the proposed method is more sensitive to the time sequence trend of propositions in temporal information fusion evaluation, and can reflect the dynamic variation trend of temporal information and the influence of time factor on the fusion evaluation. However, when the evidence remains temporally invariant, the fusion evaluation result obtained using the proposed method is less than ideal. In future work, the relationship between propositions will be introduced into evidence fusion evaluation to represent the development direction of temporal evidence.

## Data Availability

The data are available from the corresponding author on reasonable request.
